# Structure–Property Relationships of Granular Hybrid Hydrogels Formed through Polyelectrolyte Complexation

**DOI:** 10.1021/acs.macromol.3c02335

**Published:** 2024-03-20

**Authors:** Julien Es Sayed, Adrivit Mukherjee, Siham El Aani, Nayan Vengallur, Marcus Koch, Andrea Giuntoli, Marleen Kamperman

**Affiliations:** † Zernike Institute for Advanced Materials (ZIAM), University of Groningen, Nijenborgh 4, 9747 AG Groningen, The Netherlands; ‡ Engineering and Technology Institute Groningen (ENTEG), University of Groningen, Nijenborgh 4, 9747 AG Groningen, The Netherlands; § INM − Leibniz Institute for New Materials, Campus D2.2, 66123 Saarbrücken, Germany

## Abstract

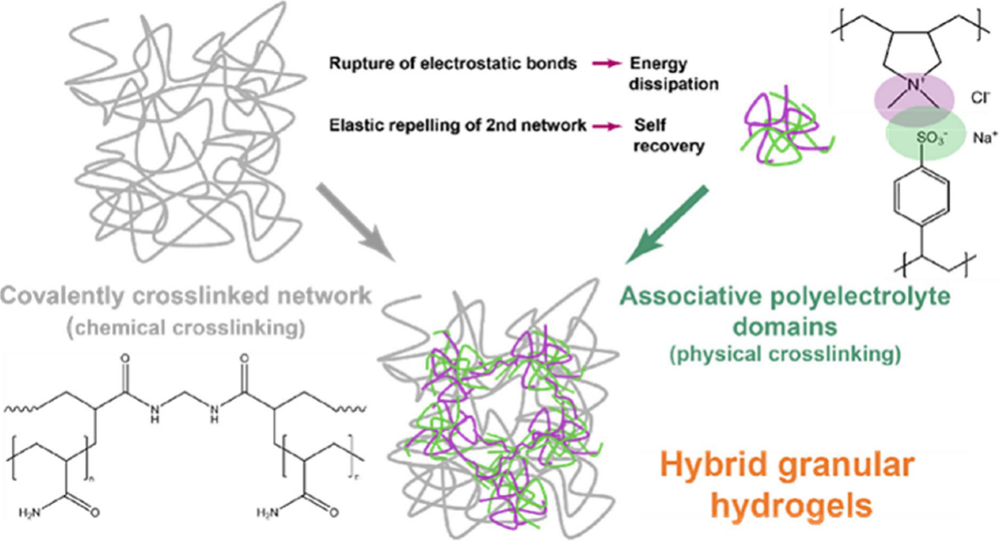

Hybrid hydrogels are hydrogels that exhibit heterogeneity in the network architecture by means of chemical composition and/or microstructure. The different types of interactions, together with structural heterogeneity, which can be created on different length scales, determine the mechanical properties of the final material to a large extent. In this work, the microstructure–mechanical property relationships for a hybrid hydrogel that contains both electrostatic and covalent interactions are investigated. The hybrid hydrogel is composed of a microphase-separated polyelectrolyte complex network (PEC) made of poly(4-styrenesulfonate) and poly(diallyldimethylammonium chloride) within a soft and elastic polyacrylamide hydrogel network. The system exhibits a granular structure, which is attributed to the liquid–liquid phase separation into complex coacervate droplets induced by the polymerization and the subsequent crowding effect of the polyacrylamide chains. The coacervate droplets are further hardened into PEC granules upon desalting the hydrogel. The structure formation is confirmed by a combination of electron microscopic imaging and molecular dynamics simulations. The interpenetration of both networks is shown to enhance the toughness of the resulting hydrogels due to the dissipative behavior of the PEC through the rupture of electrostatic interactions. Upon cyclic loading–unloading, the hydrogels show recovery of up to 80% of their original dissipative behavior in less than 300 s of rest with limited plasticity. The granular architecture and the tough and self-recoverable properties of the designed hybrid networks make them good candidates for applications, such as shape-memory materials, actuators, biological tissue mimics, and elastic substrates for soft sensors.

## 1 Introduction

Hydrogels are one of the most abundant classes of soft materials, ranging from natural origin, e.g., any biological tissue, to fully synthetic. Extensive efforts have been made to design and engineer an ever-growing range of synthetic hydrogels that meet specific requirements for applications including medical devices, drug delivery platforms, and wearable sensors.^1,2^ For many applications, the main drawback of single network hydrogels is their poor mechanical properties.^3^ In the past decade(s), it has been shown that hybrid network architectures (HNs) can be extraordinarily efficient in improving these mechanical properties.^4^

HNs are a broad family of hydrogels that can contain multiple interpenetrated networks and/or multiple components ranging from organic to inorganic fillers.^4^ To improve the mechanical properties of hydrogels, both the type and density of interactions and the structure on different length scales have been varied. Chronologically, hybrid composite hydrogels in which the organic polymer network is loaded with nano- to micron-sized particles were investigated first. In these hydrogels, the shape and the connectivity between the particles allows for controlled local inhomogeneities that directly impact the mechanical properties.^5,6^ The interactions (physisorption or chemical grafting) between polymer chains and inorganic particles enable an efficient transfer of the load through friction, debonding, or even disaggregation of the particles, thereby significantly enhancing the toughness of the resulting hydrogels. Later, the design of interpenetrated networks, such as double network hydrogels, has shown to be a very successful strategy to increase the toughness of fully organic hydrogels.^3,7,8^ In double network hydrogels, both a brittle, also named “sacrificial” network designed to break preferentially upon loading and a stretchable network act synergistically to enhance the mechanical properties. Initially, the two networks were based only on covalent bonds, which impaired the cyclability of the toughening mechanism due to the impossibility of the sacrificial network to heal after one loading–unloading cycle. As a further improvement, the use of self-healable supramolecular interactions such as hydrogen, ionic, or metal–ligand bonds to build the sacrificial networks was found to be beneficial in endowing the double network hydrogels with time-dependent recovery of the toughening properties.^8−11^ Finally, hybrid hydrogels can also be developed by combining polymer networks with drastic dissimilar water solubility.^12−14^ Usually, a preformed network template or scaffold that is manufactured with a nonwater-soluble polymer such as polycaprolactone is embedded in a network made of a hydrophilic polymer. This leads to a hybrid hydrogel with a predefined micro- to macro architecture combining the stiffness of the hydrophobic network and the deformability of the hydrophilic one. Recently, Zhang et al. reported an ingenious approach to engineer tough hybrid hydrogels with microstructural complexity through polymerization of a hydrophilic poly(acrylic acid) network inside a hydrophobic poly(ethyl acrylate) one.^15^ Upon immersion in water, microphase separation between the two networks was obtained, where the load could be successfully transferred between the two immiscible interpenetrated networks.

In light of this study, the use of liquid–liquid phase separation (LLPS) within a hydrogel matrix appears to be a promising approach to create hybrid networks with complex microarchitectures but also high toughness. In this work, we develop, for the first time, a hybrid network topology via LLPS using polyelectrolyte complexes (PEC). PECs are composed of two tightly interacting, oppositely charged polyelectrolytes.^16^ Hydrogels obtained from PEC are promising for a wide range of applications such as recyclable structural materials (fibers), films for packaging, filtration membranes, underwater adhesives, and as (bio)ink for 3D printing, to cite a few.^17−27^ The processing of a PEC material is usually enabled by addition of salt that is considered a plasticizer in an analogous way as small organic molecules or even water can be considered for polymer melts, and rubbers.^16,28−30^ Above a critical salt concentration value, the electrostatic associations between polymer chains are completely screened.^31^ Upon decreasing the salt concentration of the medium, electrostatic associations between oppositely charged polymers start to form while water is being expelled. The lower the salt concentration, the stronger the associations are, and dissociation events are slowed down considerably. The strength, dynamics, and architecture of the resulting PEC, and eventually their final mechanical properties, are highly dependent on the amount of the residual salt present in the system.^32^

Consequently, our approach is based on the interpenetration of a PEC network with a second intrinsically elastic network. The PEC network is obtained by desalting an initially homogeneous solution composed of poly(4-styrenesulfonate) (pSS) and poly(diallyldimethylammonium chloride) (pDADMAc) chains embedded in a chemically cross-linked polyacrylamide (pAAm) hydrogel network. To understand the network formation and to relate the microstructure to the mechanical properties, we performed a detailed microstructural investigation by means of electron microscopy imaging and molecular dynamics simulations. Both methods reveal the formation of a hybrid granular network (HN) architecture, irrespective of the pSS-pDADMAc content. The formation of this granular structure is attributed to the “crowding” effect exerted by the synthesized pAAm chains on the pSS-pDADMAc complex. Finally, the mechanical properties of the resulting HNs are compared to those of single pSS-pDADMAc PEC and pAAm networks.

## 2 Experimental Section

### 2.1 Materials

Poly(sodium 4-styrenesulfonate) (pSSNa) with an average molecular weight of 200 kg mol^–1^, poly(diallyldimethylammonium chloride) (pDADMAc) with an average molecular weight of 200–350 kg mol^–1^, potassium bromide (KBr), acrylamide (AAm), *N*,*N*′-methylenebis(acrylamide) (MBA), ammonium persulfate (APS), and *N*,*N*,*N*′,*N*′-tetramethylethylenediamine (TEMED) were purchased from Sigma-Aldrich (Darmstadt, Germany) and used as received. Deionized water was produced by reverse osmosis (conductivity <10 μS cm^–1^ ≈ 10^–4^ M NaCl).

### 2.2 pSS-pDADMAc PEC Material Preparation

pSSNa and pDADMAc were mixed volumetrically with a stoichiometric ratio of oppositely charged monomer units ([−SO_3_^–^] = [−NR_4_^+^] = 0.15 M) with a KBr concentration of 1.5 M. The mixture was left to mix overnight at room temperature to yield a white pSS-pDADMAc PEC precipitate. The PEC was filtered, extensively washed with water, and dried overnight in an oven at 120 °C. Then, the obtained dry pSS-pDADMAC complex with a negative-to-positive charge ratio of 1:1.08 determined by ^1^H NMR (Figure S1) was dissolved in 2.5 M KBr solution at a 20 wt v^–1^ % concentration and used as a stock solution for preparing both the single and hybrid network hydrogels. The single network pSS-pDADMAC PEC hydrogel was obtained by first casting the 20 wt v^–1^ % pSS-pDADMAC stock solution into a dog-bone mold (see Figure S2 for the dimensions), which was then immersed in a salt-free water bath to trigger the coagulation of the PEC. The coagulation bath was exchanged with salt-free water until its conductivity reached the conductivity of the reverse osmosis water used (<10 μS.cm^–1^). The dog-bone shaped samples were then used for mechanical testing and electron microscopic characterization and further named **PEC-20** standing for “PEC with 20 wt v^–1^ % pSS-pDADMAc”.

### 2.3 Hybrid Network Hydrogel Preparation

The polyacrylamide (pAAm)-based hybrid network hydrogels containing 0, 5, 10, 15, and 20 wt v^–1^ % of pSS-pDADMAc, further named **SN-0**, **HN-5**, **HN-10**, **HN-15**, and **HN-20**, were prepared as follows. AAm (1.140 g, 16.000 mmol), MBA (0.0024 g, 0.016 mmol), and APS (0.0022 g, 0.010 mmol), 0 to 4 mL of the 20 wt v^–1^ % pSS-pDADMAc stock solution, and 4 to 0 mL of a 2.5 M KBr aqueous solution were successively added in a 20 mL vial. Table S1 summarizes the quantities of reagents used for each hydrogel. Once homogeneous, the hydrogel precursor solutions were degassed with N_2_ for 5 min to remove the dissolved dioxygen. Then, 30 μL (0.2 mmol) of TEMED was added under strong stirring in the hydrogel precursor solutions to trigger the pAAm polymerization. After homogenization, the solutions were quickly injected into molds consisting of two rigid Plexiglas walls and 2 mm silicone spacers and allowed to polymerize overnight at room temperature. Then, the obtained hydrogels were carefully unmolded and immersed in a salt-free water coagulation bath to perform the desalting. The coagulation bath was exchanged with salt-free water unless otherwise stated until its conductivity reached the conductivity of the reverse osmosis water used (<10 μS cm^–1^) and the swelling equilibrium of the hydrogels was attained. The resulting samples were further punched into a dog-bone shape (see Figure S2 for the dimensions) for further mechanical testing.

### 2.4 Swelling Equilibrium of the Hydrogels

The volume swelling equilibrium of the hydrogels *Q*_V_, was determined as a function of the immersion time in the coagulation bath, following the eq 1:
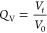
1where *V*_0_ is the volume of the cylindrical-shaped hydrogel before immersion, and *V*_*t*_ is the volume of the hydrogel *t* hours after immersion.

### 2.5 Tensile Testing Until Failure

Uniaxial tensile tests were performed on the swollen dog-bone-shaped hydrogels in air using a tensile tester (Instron 5565, load cell of 10 N). No extensive drying of the samples during the measurements was observed, as the mass of the samples remained unchanged (no more than 1% weight loss by water evaporation). All the samples were strained at a velocity of 30 mm min^–1^ (strain rate of 1.5 min^–1^). The ultimate stress and fracture strain was defined as the nominal stress and strain at the breaking point, respectively. Young’s modulus was defined as the initial slope of the stress–strain curves. Strain energy density was defined as the area under the stress–strain curve. The reported values are reported as the mean value ± SD of measurements performed in triplicate.

### 2.6 Cyclic Tensile Testing

The cyclic loading tensile tests for evaluating the recovery efficiency of the single and hybrid network hydrogels were performed using a CellScale UniVert S tensile tester. The hydrogels were stretched to ε = 0.5 mm.mm^–1^ at a velocity of 30 mm min^–1^ at room temperature. Then, the samples were returned to the initial displacement immediately at the same speed. After each loading/unloading test, the samples were left to stand at room temperature from 0–1800 s. The energy dissipation *E*_diss_ was estimated from the hysteresis area following the eq 2:
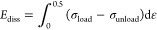
2where σ_load_ and σ_unload_ are the stress during loading and unloading, respectively. The recovery efficiency (%) was calculated by dividing the *E*_diss_ value of the sample at the given rest time by one of the virgin samples. The residual strain was determined as the strain for which the stress reached a value of 0 after the unloading cycle. The reported values are reported as mean value ± SD of measurements performed in triplicate.

### 2.7 Creep-Recovery Measurements

The creep-recovery experiments were performed using a CellScale UniVert S tensile tester. The evolution of the residual strain of the hydrogel samples as a function of the rest time (*t*) after one loading–unloading cycle ε = 0.5 mm.mm^–1^ was measured by monitoring the normal force at 0 for 500 s.

### 2.8 Transmission Electron Microscopy (TEM)

TEM images were recorded on a JEOL JEM-2100 LaB_6_ transmission electron microscope at 200 kV acceleration voltage via a Gatan Orius SC1000 camera in bright field mode at a sample temperature of 19 °C. The hydrogel samples were obtained by biopsy punching. A tweezers was used to extract a small piece of the hybrid network hydrogels from the bulk. Then, a holey carbon TEM grid (Plano, Wetzlar, type S147-4) was placed between the two arms of the tweezers. The tweezers were closed, and the small piece of hydrogel formed a thin film on the holey carbon film.

### 2.9 Environmental Scanning Electron Microscopy (ESEM)

ESEM images were recorded on an FEI Quanta 400 ESEM FEG. The hydrogel samples were freshly cut under wet conditions using a blade and placed on the precooled Peltier stage at 3 °C of the ESEM with the wet cross-section on top. ESEM investigation was started at 3 °C and 800 Pa; then, the pressure was gradually decreased to 700 Pa to evaporate the excess water on the surface of the hydrogels and reveal the microstructure.

### 2.10 Molecular Dynamics Simulation

The HN-5 hydrogel was simulated with molecular dynamics using a standard coarse-grained FENE model^33^ with Lennard-Jones beads of size σ, interactions strength ϵ_LJ_, and cutoff *r*_c_ = 1.12σ to simulate good solvent conditions. The experimental molecular weights for pSS and pDADMAc correspond to ∼1000 monomers, and two tetravalent monomers in the pAAm network are spaced ∼300 monomers apart. Typically, one model bead would correspond to ∼2 monomers (or 1 Kuhn length),^34^ but this would make the simulations computationally too expensive, so we scaled down all molecular weights by a factor of 10 to qualitatively capture the behavior of the hydrogel. With this in mind, pSS and pDADMAc were modeled as linear chains consisting of 50 beads, each carrying 1 charge. They also have an angular potential *U*_angle_ = *K*_θ_ [1 + cos (θ)], *K*_polyele_ = 4 to incorporate, via moderate chain stiffness, the effect of entanglements present experimentally and lost in our scaled-down model. Charged beads interact with a Coulomb potential . The pAAm network is modeled by cross-linking telechelic stars with four arms of 7 beads each, which simplifies and speeds up the pAAm polymerization process in the model, maintaining a similar final network structure. Salt ions are represented by single beads of charge 1 and the solvent is modeled implicitly. All beads in the model have the same mass *m* and size σ. The size of salt ion beads is chosen for simplicity and in line with recent literature,^35^ considering that theoretical studies have shown that the size of the salt beads does not have a large effect on the thermodynamics of the coacervation process.^36^ The long-range Coulomb interactions are calculated by the particle–particle particle-mesh (PPPM) method in LAMMPS with a target accuracy of 1 × 10^–4^. The positions and velocities of particles are updated by a velocity-Verlet algorithm with a time step of 0.005τ, where τ = (*m*σ^2^/*k*_B_*T*)^1/2^. The thermal energy is set to *k*_B_*T* = 1.0. Similarly, to the HN-5 hydrogel, the simulation has a 7% volume concentration of pAAm monomers and a 3% volume concentration of pSS-pDADMAc chains, for a total of 240 polyelectrolyte chains and 1000 4-arm stars. Salt ions corresponding to the critical salt concentration are then added to the system (see Figure S4 in the Supporting Information for more information on how it was calculated). The system was first equilibrated in the NVT ensemble using a Nose-Hoover thermostat for 3 × 10^4^τ, without considering the Coulomb interactions from charged monomers to initialize a homogeneous solution. After equilibration, we simultaneously turned on the Coulomb interactions between charged monomers (both polyelectrolyte beads and salt ions) and completed the formation of the pAAm network by cross-linking the arm ends of the stars when they were closer than 1.12σ.^37^ No intramolecular bonds are allowed to minimize loops and network defects. A degree of curing of 98% was reached at the end of the process. After equilibration of the system, we start desalting by removing an equal number of cations and anions. For every 5 τ_LJ_, 400 pairs of salt ions were randomly chosen and deleted. The process was continued until all salt ions were removed. The system was then allowed to reach a steady state. We quantified the extent of coacervation by measuring the surface area of the coacervate accessible to the solvent. This is calculated by considering a concentric sphere of radius *r*_s_ =1.3σ around each polyelectrolyte bead. Then, the area of sections of this sphere that do not overlap with other such spheres was calculated and summed. When all the polyelectrolyte chains are well separated, this results in a total area approximately equal to *A* ≈ 4π*r*^2^ × *N*_polyele_ ≈ 2 × 10^5^σ^2^. Our results are then reported as a fraction of this maximum value.

### 2.11 ^1^H NMR

^1^H NMR experiments in D_2_O were performed on a Bruker Avance III HD spectrometer operating at 400 MHz, using a standard 5 mm broadband Smart probe regulated at 25 °C. The chemical shift in parts per million from tetramethylsilane was referenced to the residual isotopomer solvent signal (HOD). 32 scans were performed for each measurement.

## 3 Results and Discussion

### 3.1 Hybrid Network Hydrogel Synthesis

**Figure 1:**
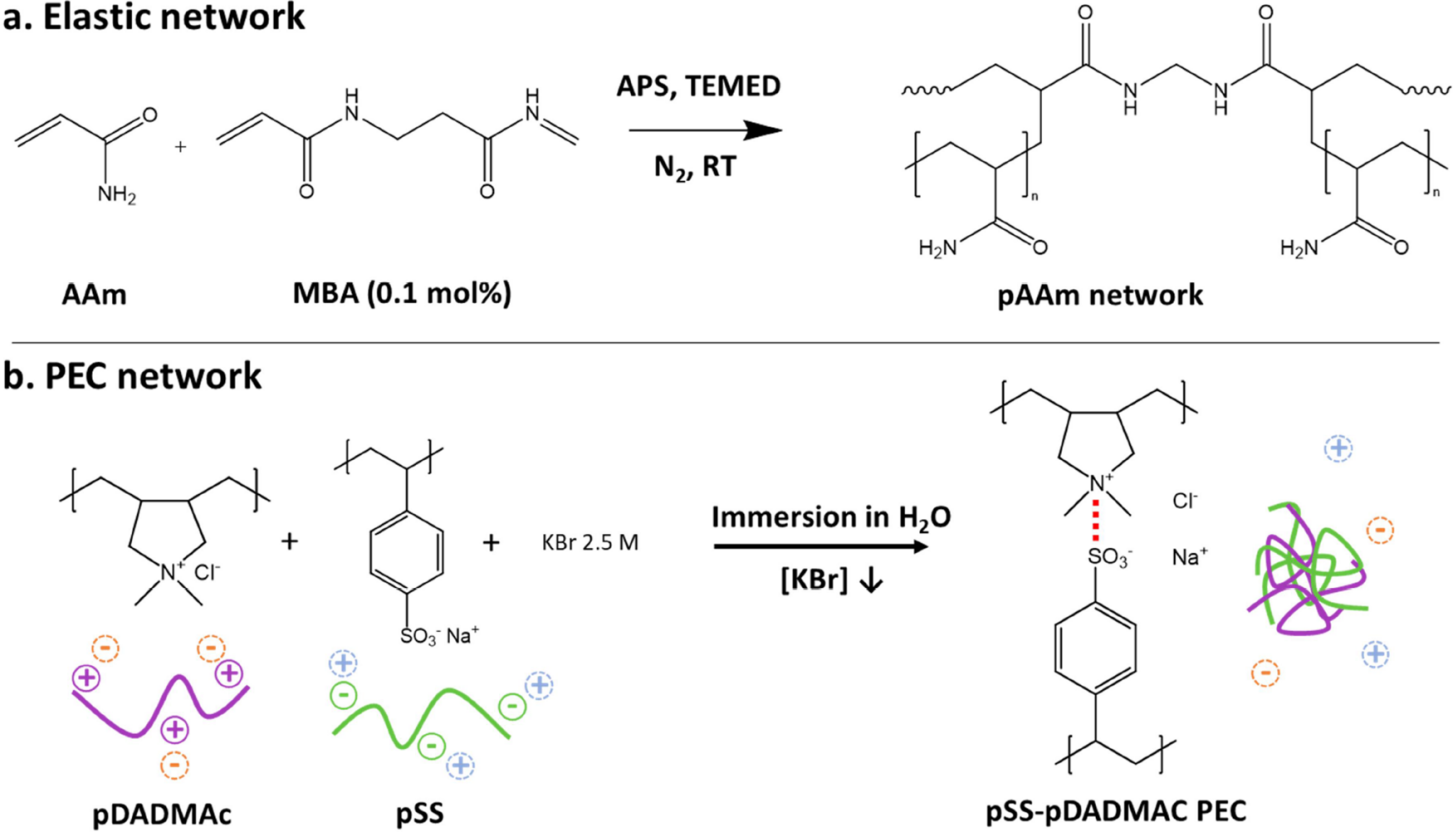
(a) Synthesis scheme of the pAAm elastic network, and (b) procedure of the pSS-pDADMAc PEC network formation.

The hybrid network hydrogels were obtained through a two-step fabrication route schematically shown in Figure 1 and detailed in the experimental section of the manuscript. Briefly, the elastic polyacrylamide (pAAm) network was first obtained by free radical polymerization using *N*,*N′*-methylenebis(acrylamide) (MBA) as a chemical cross-linker. The polymerization was performed in a 2.5 M KBr aqueous solution and with 0, 5, 10, 15, or 20 wt v^–1^ % of dissolved pSS-pDADMAc chains using a 1 to 1.08 molar ratio of negative to positive repetitive units (Figure S1). After gelation of the pAAm network, the hydrogels were immersed in a salt-free coagulation bath to extract KBr from the sample and trigger the electrostatic complexation between negatively charged pSS and positively charged pDADMAc chains. In such a way, the decrease in the ionic strength through the diffusion of the salt out of the matrix enables the formation of an electrostatic-based pSS-pDADMAc PEC network inside the pAAm hydrogel matrix. The synthesized samples will be further named SN-X and HN-X, with “SN” standing for “single network” (pAAm only), “HN” standing for “hybrid network,” and “X” standing for the pSS-pDADMAc wt v^–1^ % (0, 5, 10, 15, or 20) introduced. As a means of reference, the PEC-20 single network (starting with pSS-pDADMAc at a 20 wt v^–1^ % concentration) was also prepared.

### 3.2 Hybrid Network Structure Formation: Polymerization of the pAAm Network

**Figure 2:**
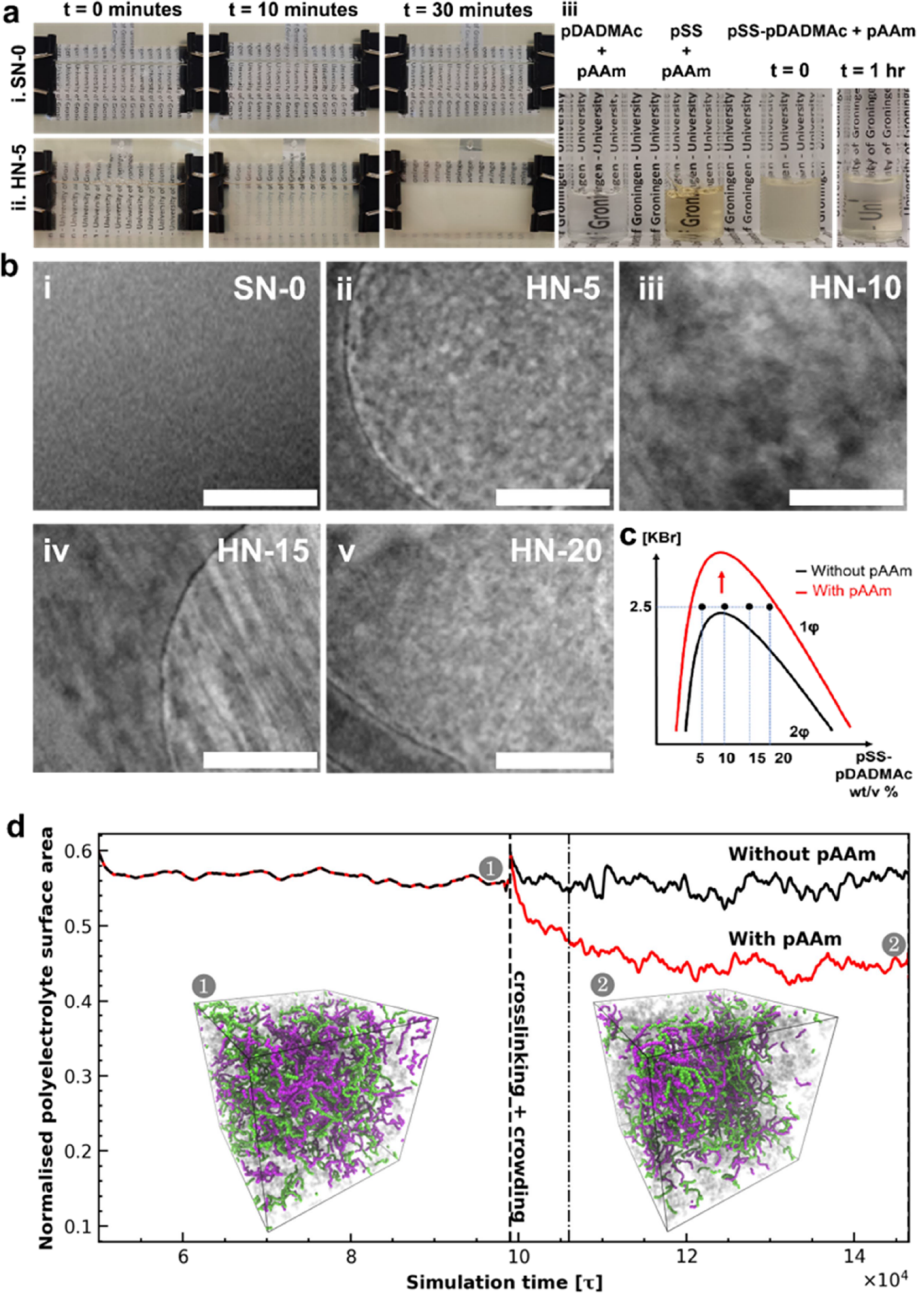
(a) Images of the (i) SN-0 and (ii) HN-5 hydrogels before *t* = 0, 10, and 30 min after starting the polymerization of the pAAm network. (iii) Mixing experiments were used to investigate the “crowding” effect of the pAAm chains in a pSS-pDADMAC mixture in 2.5 M KBr. pAAm linear chains were added to either a pSS solution, a pDADMAc solution or a pSS-pDADMAC solution. Only the last mixture leads to a microscopic phase separation with, eventually, a complete macroscopic phase separation after 1 h rest. (b) Transmission electron microscopy images of the (i) SN-0, (ii) HN-5, (iii) HN-10, (iv) HN-15, and (v) HN-20 hydrogels. The scale bars are 500 nm. (c) Qualitative phase diagram proposed for the complex coacervation of pSS-pDADMAC mixtures in the presence or absence of pAAm chains. The two-phase region where electrostatic complexation between pSS and pDADMAc occurs enlarges due to the “crowding” effect exerted by the pAAm chains. (d) Molecular dynamics simulation depicting the HN-5 hydrogel system (1) in a baseline homogeneous solution with no Coulomb interactions and (2) after completion of the polymerization of the pAAm network and considering Coulomb interactions between charged beads to start the complexation process. The same “crowding” effect is observed: weak complexation and formation of heterogeneous pSS-pDADMAC filaments occurs even above the critical salt concentration when the pAAm network is present. The second dotted line indicates the conclusion of pAAm cross-linking.

First, we investigated the mechanisms of formation of the hybrid network hydrogels and their resulting microstructure. Upon polymerization of the pAAm network, the first striking difference between the SN-0 and HN-X samples could be observed. Figure 2a-i shows the appearance of the SN-0 hydrogel, and Figure 2a-ii of the HN-5 hydrogel, which is representative of the other HN-X hydrogels. While the SN-0 hydrogel remained fully transparent, the HN-X samples transitioned from slightly yellowish transparent solutions to turbid hydrogels, whose opacity increased with the pSS-pDADMAc content. To investigate the underlying mechanism leading to these differences, the microstructure of the hydrogel samples, just after polymerization of the pAAm network, was imaged by TEM. In Figure 2b-i, we observe that the SN-0 hydrogel shows a monotonic surface with no distinctive inhomogeneity at the nanoscopic scale. In striking contrast, all of the HN-X samples revealed a phase-separated granular-like structure (Figure 2b-ii–v). An energy-dispersive X-ray spectroscopy (EDX) analysis (Figure S3) highlighted that the phase-separated granules (dark domains) were enriched in a sulfur element that can originate from only the sulfonate moiety of the pSS chains. However, the possible presence of pDADMAc is impossible to reveal by this technique as it contains the same atoms as pAAm. Nevertheless, control experiments involving the mixing of pSS, pDADMAc, and pAAm linear chains in a 2.5 M KBr aqueous solution revealed that only the quaternary mixture (pSS-pDADMAc-pAAm-water-2.5 M KBr) led to a turbid phase-separated solution (Figure 2a-iii). Eventually, the system fully phase-separated into two liquid phases. For all of the other combinations, a homogeneous solution was obtained. This set of results strongly points toward the formation of a liquid-like pSS-pDADMAc complex coacervate upon mixing with neutral pAAm chains.^38,39^ It is worth noting that the salt concentration in the medium was maintained at 2.5 M KBr, which was initially high enough to screen the attractive interactions between pSS and pDADMAc chains. However, the presence of linear pAAm chains induced an additional attractive potential between the oppositely charged polyelectrolyte chains, which triggered the LLPS increasing the two phases region, as schematically shown in Figure 2c. This so-called “crowding” phenomenon has already been reported in the literature for complex coacervate systems, mainly using linear poly(ethylene oxide) (PEO) chains.^40,41^ It arises from the competition for hydration between the water-soluble neutral pAAm chains and the polyelectrolyte chains.

In the case of the hybrid networks, homogeneously distributed turbidity was observed for all the hydrogels for at least 1 week instead of macroscopic phase separation. We hypothesize that the pSS-pDADMAc complex coacervate droplets, originally formed upon polymerization of pAAm chains, remain trapped by the concomitant formation of the chemically cross-linked pAAm network. To confirm this hypothesis, we performed a molecular dynamics simulation to follow the phase separation process inside the pAAm network. In Figure 2d, we measured the overall surface of the polyelectrolyte chains accessible to the solvent as a function of simulation time τ during the polymerization of the pAAm network to quantify the degree of electrostatic complexation in a coarse-grained model. This surface area has a maximum of ∼2 × 10^5^, when all of the polyelectrolyte chains are well separated and only in contact with the solvent. The surface area is represented as a fraction of this theoretical maximum. We started with a homogeneous solution where Coulomb interactions of charged beads are not calculated and the pAAm network (if present) is not fully cross-linked, which we used as a baseline measurement. We then introduced the effect of Coulomb interactions, and simultaneously completed the cross-linking of the pAAm network (starting from the dotted line in Figure 2d). The decrease in the polyelectrolyte surface area when the chains are embedded in the pAAm network reveals a similar “crowding”-induced LLPS above the critical salt concentration of the pSS-pDADMAc mixture with a polymer concentration identical to the one of the HN-5 hydrogels. This coacervation phenomenon is also directly visible in the provided simulation snapshots in Figure 2d. Consistent with the experiments, upon activation of the Coulomb interactions and polymerization of pAAm (dotted line), the pSS-pDADMAc chains locally and weakly coacervate, forming heterogeneous filaments across the pAAm network. It is essential to notice that this electrostatic complexation phenomenon does not happen at the same polyelectrolytes and salt concentrations in the absence of the pAAm network (black curve in Figures 2d and S4).

### 3.3 Hybrid Network Structure Formation: Desalting Process

**Figure 3:**
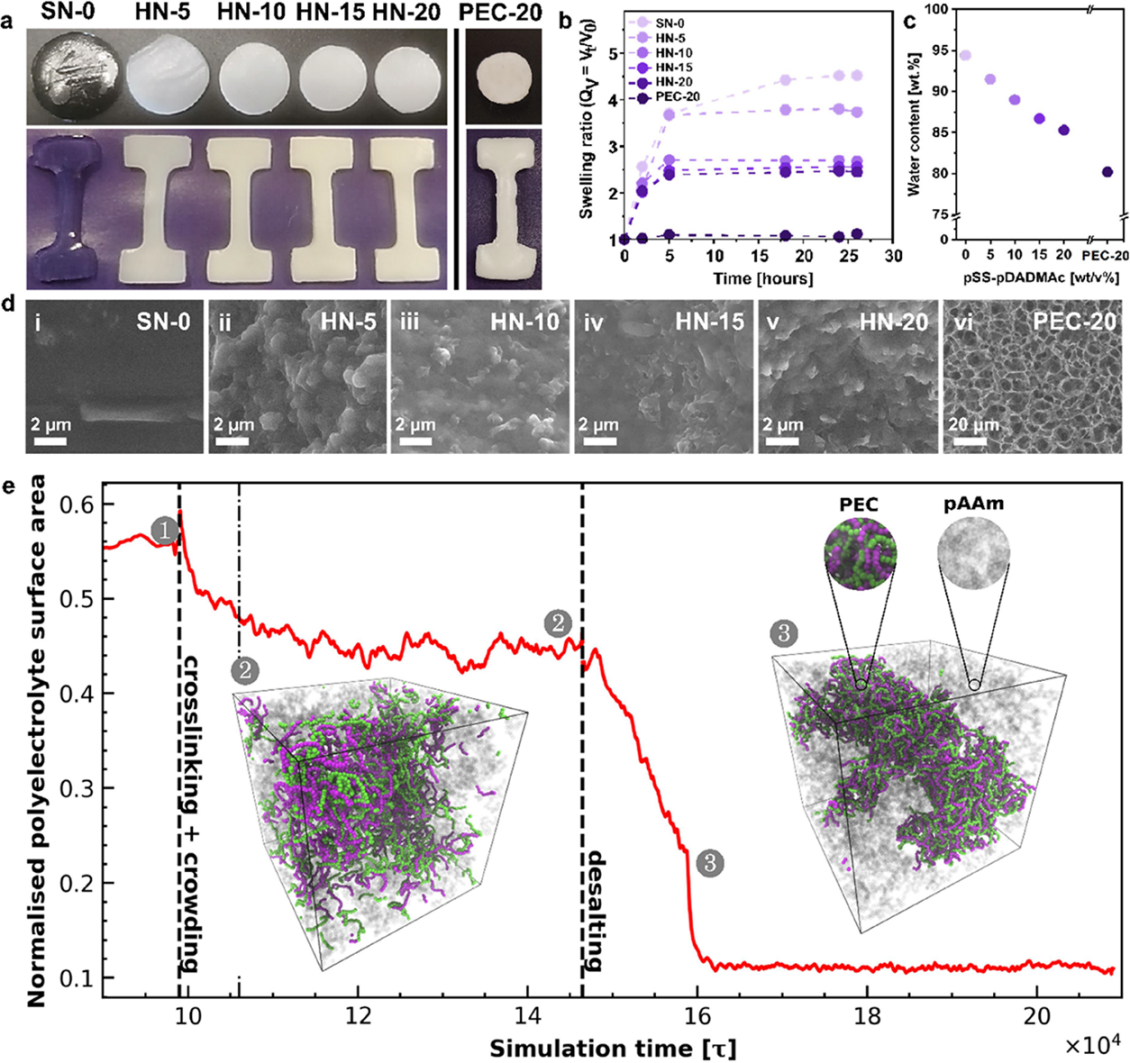
(a) Image showing the hydrogels after desalting and swelling in a salt-free bath. (i) Cylindrical samples used for swelling experiments and (ii) dog-bone shaped samples cut-out after swelling equilibrium is attained and used for mechanical characterization experiments. (b) Volume swelling ratio (*Q*_V_) as a function of time and (c) water content of the hydrogels after the equilibrium volume ratio was attained. (d) Environmental scanning electron microscopy (ESEM) images of the (i) SN-0, (ii) HN-5, (iii) HN-10, (iv) HN-15, (v) HN-20, and (vi) PEC-20 hydrogels. The scale bars are 2 μm, except for the PEC-20 hydrogel for which the scale bar is 20 μm. (e) Molecular dynamics simulation depicting the HN-5 hydrogel system upon desalting. The local degree of coacervation increases with tightening of the complex coacervate filaments dispersed in the pAAm network upon desalting. Snapshots of the system (2) before and (3) after desalting and swelling.

After polymerization of the pAAm network, the hydrogels were immersed in consecutive salt-free water baths to desalt and strengthen the electrostatic interactions between pSS and pDADMAc. Instantaneously, after immersion, the HN-X samples all turned white and opaque, while the SN-0 remained transparent (Figure 3a). During the desalting process, the hydrogels underwent swelling, which was measured and reported in Figure 3b, and the equilibrium water content was reported in Figure 3c. The equilibrium volume swelling ratio was reached for all hydrogels before 18 h of immersion. The highest swelling ratio was observed for the SN-0 hydrogel (*Q*_V_ = 4.5 and water content of ≈94 wt %). Upon increasing the pSS-pDADMAc content in the hybrid network hydrogels, the equilibrium swelling ratio decreased from 3.7 (92 wt % water) to 2.4 (85 wt % water) for the HN-5 and HN-20, respectively. In striking contrast, the pure pSS-pDADMAC PEC hydrogel, PEC-20, only very moderately swelled (*Q*_V_ = 1.1, 80 wt % water), even after prolonged immersion. The high swelling capacity of the SN-0 pAAm hydrogel in water is commonly observed and can be well explained and predicted by the Flory–Rehner theory.^42^ While the high affinity of the pAAm for water tends to swell the hydrogel infinitely, the presence of chemical cross-links (0.1 mol % MBA vs AAm here) gives rise to an elastic contribution limiting the swelling of the hydrogel to an equilibrium. On the other side of the swelling spectrum, the very low swelling ratio of the PEC-20 hydrogel can be related to the rapid formation of strong and relatively nondynamic electrostatic interactions between pSS and pDADMAc chains.^43^ Indeed, the combination of a high density of consecutive negative–positive electrostatic pairs and chain entanglement at such polymer concentration (20 w/v %) formed upon immersion in salt-free water induces the formation of a stiff hydrogel whose characteristics and structural dimensions are frozen.^44^ In such conditions, diffusion of water and salt outward and water inward are still possible but without altering the dimension of the hydrogels. Interestingly, it appears that the equilibrium swelling ratio of the HN-X hydrogels results in a balance between the high swelling power of the pAAm network and the low swelling power of the pSS-pDADMAc network. This result is a valuable hint toward the formation of an interpenetrated network architecture upon desalting and swelling.

To gain a better insight into the nano- to microstructure of the resulting hybrid network hydrogels, ESEM imaging was used to image a freshly cut surface of the swollen hydrogels (Figures 3d-i–vi and S5). As expected, the SN-0 surface remained smooth after the desalting process,^45^ while all the HN-X hydrogels exhibited a rough surface with interconnected granules. The microstructure was observed to be drastically different from the PEC-20, pSS-pDADMAC network, which exhibited a microporous structure after desalting as has also been reported for similar systems.^16,22,46^ This porous structure is the result of internal constraints where, upon desalting, the polymer chains tend to associate strongly while water is being expelled. However, due to the relatively high polymer concentration, the diffusion of water is drastically slowed down by the usual formation of a few micrometers thick dense skin layer also observed on our sample (Figure S6).^22,23^ Thus, the phase separation process occurs at a micrometer scale within the bulk PEC material. On the contrary, the granular aspect of the HN-X samples is most likely due to the densification of the preformed pSS-pDADMAc domains under the action of the “crowding” effect of the pAAm chains composing the first network. By observation of the electron microscopic images, the granules seem to be connected, thus forming a continuous PEC network within the pAAm network. To support this hypothesis, the molecular dynamics simulations were continued, and the desalting process was followed for the HN-5 model system and presented in Figure 3e. Upon desalting, the filaments formed during the “crowding” phase tighten, and the degree of complexation increases, as measured by the surface of the PEC accessible to the solvent and observed in simulation snapshots. The heterogeneous PEC filaments are interpenetrated in the pAAm network at the end of the desalting process. After an initially kinetically trapped phase, we eventually observe complete phase separation of the model PEC, which we expect to be caused by the small size of the simulations and the short, coarse-grained polyelectrolyte chains. Indeed, from implicit salt simulations, we see that in the entanglement regime of the polyelectrolyte chains (>100–150 for this model), this arrested phase is more stable, as observed in the experiments (Figure S7). However, these more extensive scale simulations with the explicit salt needed to capture the “crowding” effect are costly, and we plan to characterize this effect more quantitatively in our future work.

### 3.4 Mechanical Properties of the Hybrid Network Hydrogels

**Figure 4:**
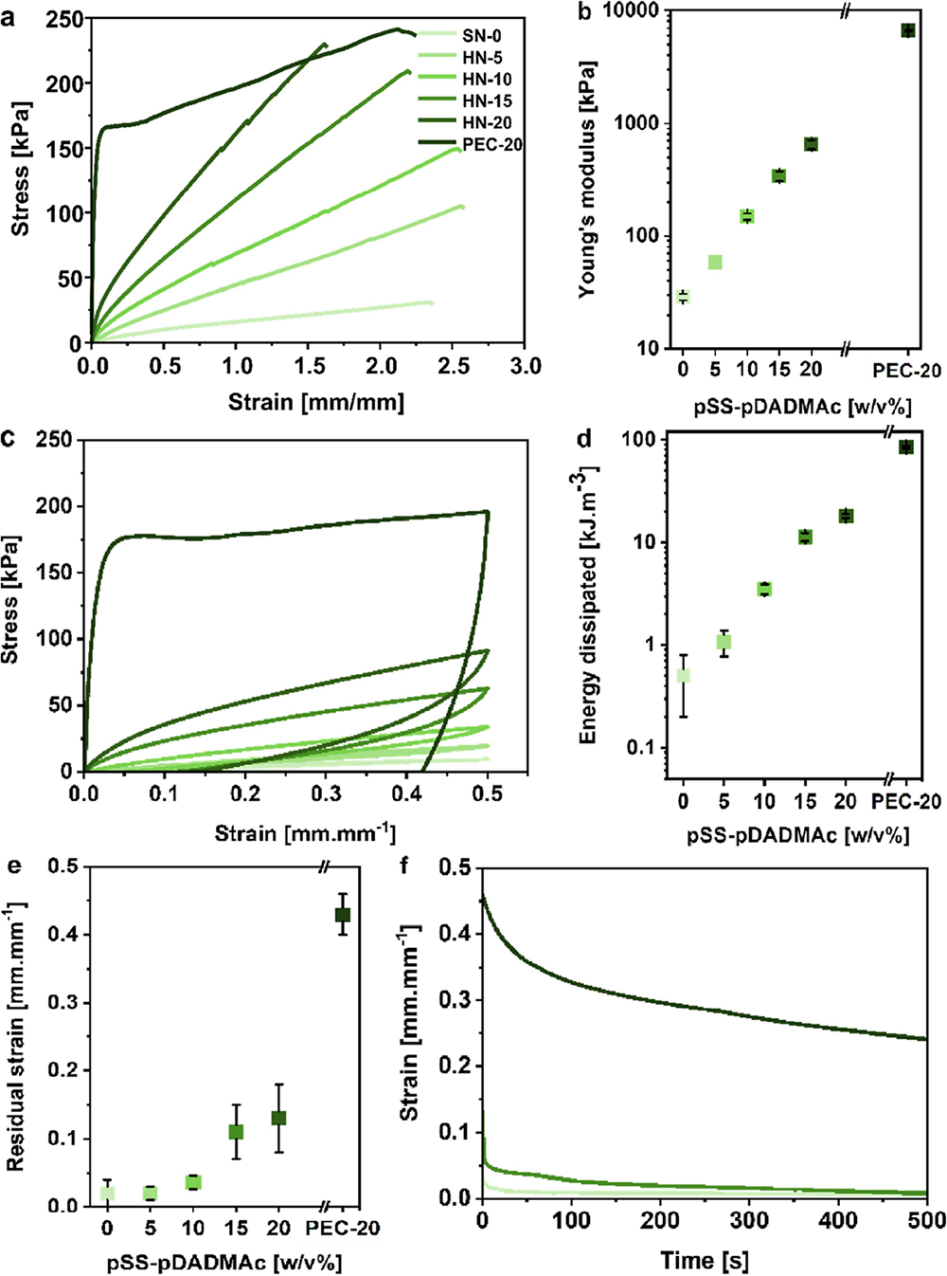
Mechanical characterization of the SN-0, HN-X and PEC-20 hydrogels. (a) Stress–strain curves obtained by tensile testing until failure. (b) Young’s modulus of the hydrogels. (c) Stress–strain curves obtained by cyclic loading–unloading testing until a strain of 0.5 mm.mm^–1^. (d) Energy dissipated and (e) residual strain after one loading–unloading cycle, as a function of the pSS-pDADMAc content. (f) Creep-recovery from 0.5 mm.mm^–1^ applied strain monitored over 500 s.

After analysis of the microstructure of the synthesized hydrogels, their mechanical properties were investigated through tensile testing, both until failure and upon cyclic loading. First, the hydrogels were subjected to uniaxial tensile stretching at a fixed strain rate of 1.5 min^–1^ until failure. The resulting stress–strain curves were recorded and are presented in Figure 4a. In agreement with the extensively reported behavior of PEC materials, we could observe that the pure PEC network, PEC-20, showed a ductile behavior with a yield strain value less than 0.1 mm.mm^–1^ and a steady increase of the stress until an ultimate strain value of around 2.2 mm.mm^–1^.^32,47^ Additionally, the PEC-20 hydrogel was relatively stiff, with a Young’s modulus of around 6.6 MPa (Figure 4b). In contrast, the pure pAAm network (SN-0) showed a classical elastic behavior, characteristic of soft hydrogels, arising from the entropic elasticity of the stretched chains within the network.^48^ The SN-0 had a Young’s modulus of around 28 kPa and could be stretched until a strain of around 2.4 mm.mm^–1^. Interestingly, considering the HN-X samples, all of the stress–strain curves exhibited a shape similar to that of the elastic SN-0 sample, with a gradual increase of the measured stress until failure. No plastic plateau or yield strain could be detected. Nevertheless, the measured Young’s moduli were observed to significantly increase with the pSS-pDADMAc content from 58 to 650 kPa, for the HN-5 and HN-20 hydrogels, respectively (Figure 4b). From this perspective, the importance of the stiff pSS-pDADMAc network on the properties of the resulting HNs could also be evidenced. It is worth noticing that the ultimate strength of the samples and the strain energy density increased concomitantly, while the maximum strain decreased to a value of 1.5 mm.mm^–1^ for the HN-20 hydrogel (Figure S8a–c). As an intermediate conclusion, the pAAm network seems to control the mode of deformation of the HN hydrogels even at high strain values, being relatively more elastic than plastic. In contrast, the pSS-pDADMAc network gives the hydrogels their stiffness.

To further confirm the mode of deformation of the hybrid network hydrogels and assess the toughening ability of the PEC network, the hydrogels were subjected to one loading–unloading cycle until a strain of 0.5 mm.mm^–1^. In Figure 4c, the highly ductile behavior of the PEC-20 hydrogel was also evidenced by the extended plastic plateau and by a residual strain measured after unloading as high as 0.43 mm.mm^–1^ (Figure 4d). Besides this, a pronounced hysteresis between the loading and unloading curve was witnessed for the dissipative behavior of the pSS-pDADMAc PEC network with a toughness, *E*_diss_, calculated to be around 85 kJ m^–3^ (Figure 4e). As identified before, the SN-0 pAAm network showed the characteristics of a purely elastic hydrogel with superimposed loading and unloading curves, evidencing the absence of a significant dissipative mechanism (*E*_diss_ < 0.5 kJ m^–3^, Figure 4e). Concerning the HN-X hydrogels, the dissipative behavior arising from the PEC network appeared to increase drastically with the pSS-pDADMAc content from *E*_diss_ = 1 kJ m^–3^ for HN-5 to *E*_diss_ ≈ 18 kJ m^–3^ for HN-20 (Figure 4e). The residual strain measured after the unloading cycle remained negligible for the HN-5 and HN-10 hydrogels (below 0.03 mm.mm^–1^) but increased up to 0.11 and 0.13 mm.mm^–1^ for the HN-15 and HN-20 hydrogels, respectively (Figure 4d). As reported in the literature, the dissipative mechanism in the PEC network arises from the disruption of the electrostatic pairs (here the sulfonate, −SO_3_^–^, and ammonium, −NR_4_^+^, moieties) between the strongly associated polyelectrolytes.^43,49^ A high density of electrostatic associations, or consecutive electrostatic pairs, results in higher energy dissipation. This can explain why the highest toughness is observed for the pure PEC network (PEC-20) and why the toughness of the HN hydrogels continuously decreases with decreasing pSS-pDADMAc content. While the presence of the pAAm network seems to impair the dissipative behavior of the hydrogels, probably by reducing the density of electrostatic associations, its elastic nature efficiently reduces the plasticity of the hybrid network hydrogels. The elastic repelling force exerted by the pAAm matrix on the PEC network minimizes residual plastic deformation even for strains up to 0.5 mm.mm^–1^, immediately after unloading. The influence of the elasticity of the pAAm network was further investigated by following the evolution of the residual strain as a function of the rest time after one loading–unloading cycle through creep-recovery experiments (Figure 4f). Remarkably, while the PEC-20 hydrogel still suffered from a residual strain value of 0.25 mm.mm^–1^ even after 500 s rest, the HN-15 and HN-20 hydrogels fully recovered their original dimensions within only 300 s rest. Such a fast residual strain recovery for a hydrogel with a multiple network architecture for which the sacrificial network is supramolecular is unusual as most of the reported systems either never fully recover their original dimensions or not before 30 min of rest.^9,10,47,50,51^ Only very recently, Yasui et al. reported a double network hydrogel in which the sacrificial network is a thixotropic hydrogel made of an oligomeric electrolyte gelator where no residual strain was observed after one loading–unloading cycle.^52^

### 3.5 Self-Recovery of the Hybrid Network Hydrogels

**Figure 5:**
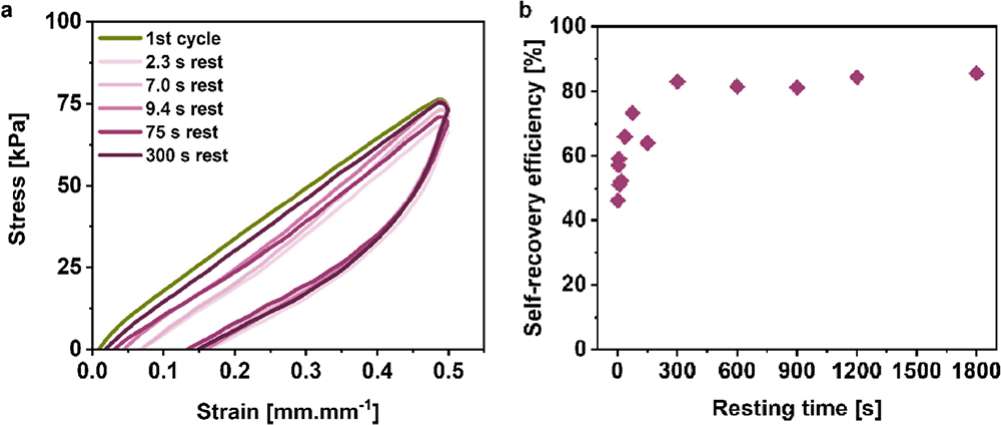
(a) Cyclic loading–unloading test of the HN-20 hydrogel after various rest times. (b) Self-recovery efficiency of the HN-20 hydrogel as a function of the rest time.

We investigated the self-recovery properties as a function of the rest time between two consecutive loading–unloading cycles. In Figure 5a, we observed that all of the samples partially recovered the hysteresis between the loading and unloading curves compared to the first cycle. The self-recovery efficiency was then further calculated by integrating the hysteresis area and reported in Figure 5b. Strikingly, we estimated that 46% of the energy dissipated by the pSS-pDADMAc network immediately recovered. The self-recovery eventually reached a plateau just above 80% after only 300 s of rest. Similar to the residual strain recovery reported earlier, this self-recovery time is one of the fastest reported in the literature regarding multiple network architecture based on a supramolecular, healable sacrificial network.^8,9,53,54^ It also highlights the potential of using tightly electrostatically associated polyelectrolytes within PEC as sacrificial and fast healable networks to design tough hydrogels with an enhanced lifetime.

### 3.6 Effect of the Bath Salinity on the Mechanical Properties of the Hybrid Network Hydrogels

**Figure 6:**
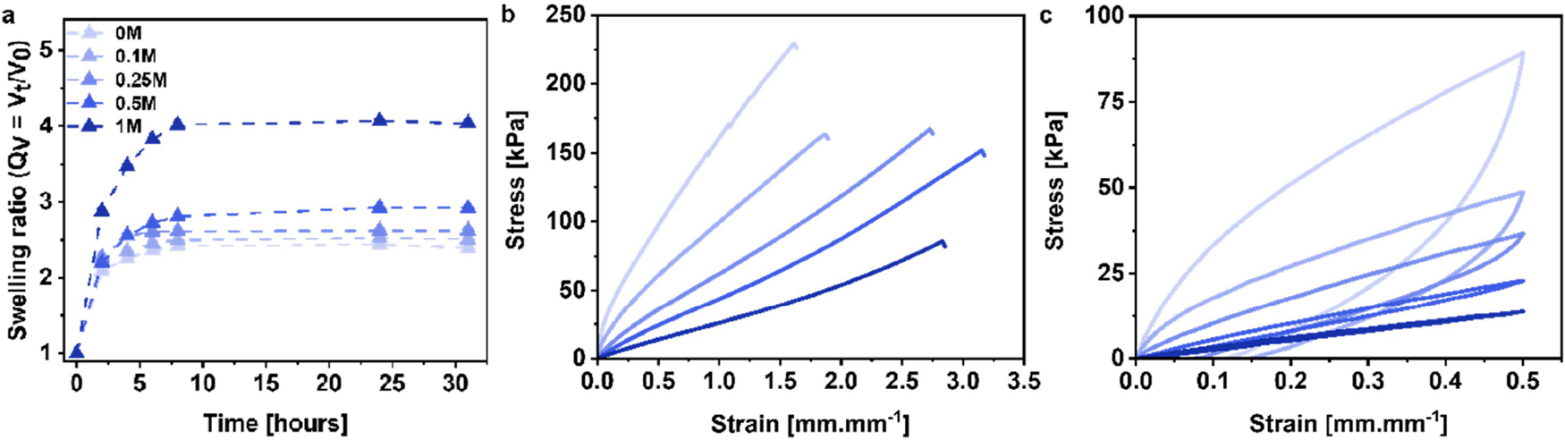
(a) Volume swelling ratio (*Q*_V_) as a function of time of the HN-20 hydrogels after immersion in bath with KBr concentrations ranging from 0 to 1 M. Stress–strain curves obtained for (b) by tensile testing until failure and (c) by cyclic loading–unloading testing until a strain of 0.5 mm.mm^–1^ for the corresponding hydrogels.

**Figure 7:**
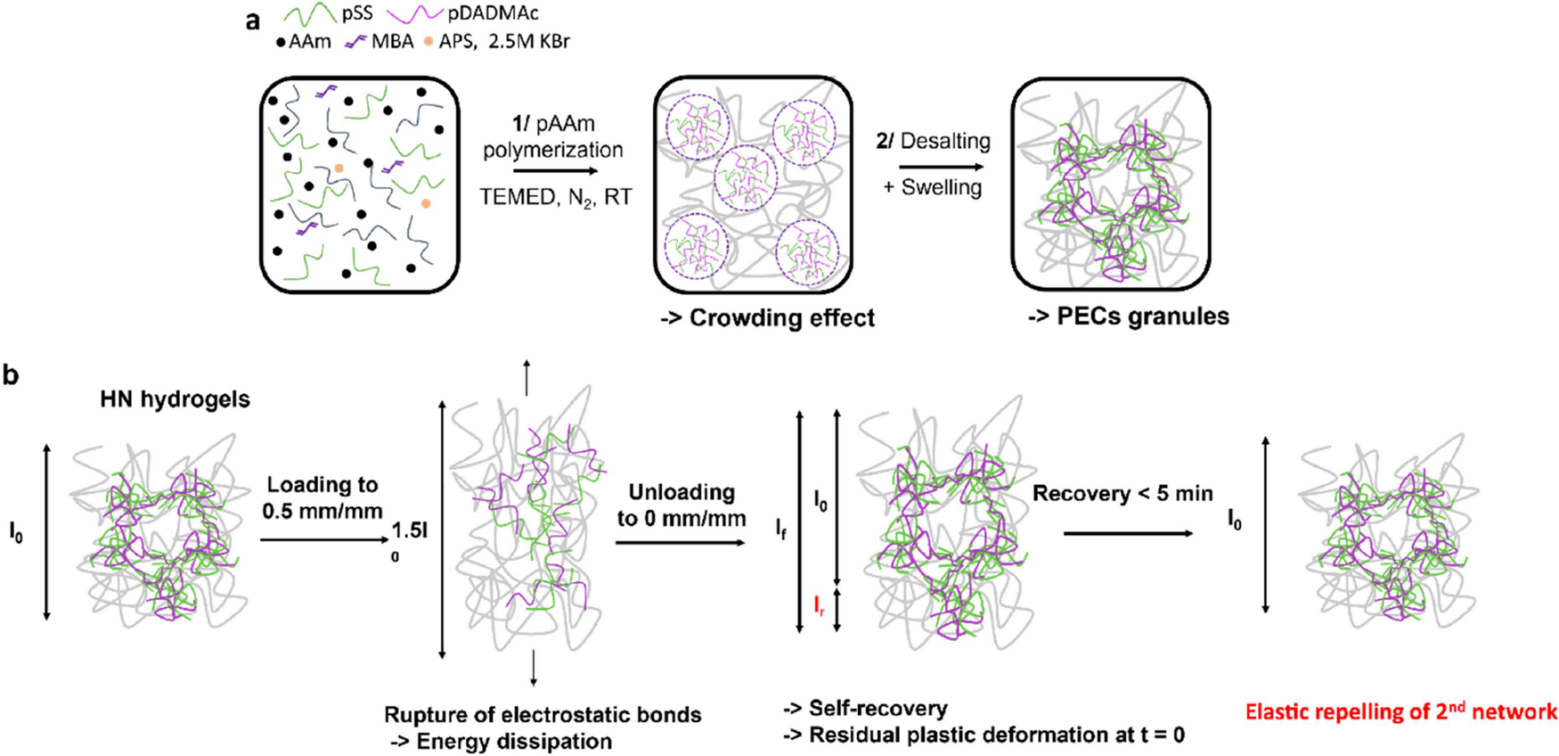
Molecular and microstructural picture (a) of the formation, and (b) of the toughening and self-recovery mechanisms of the HN hydrogels.

As already introduced, PEC materials are well known to be efficiently plasticized by salt ions.^16,31,49^ Therefore, we investigated the effect of immersing the hybrid network hydrogels after pAAm polymerization in an aqueous bath with various KBr concentrations, namely, 0, 0.1, 0.25, 0.25, and 1 M. The results obtained for the HN-20 only are discussed here, and the different samples are named as follows: HN-20/X, with X being the KBr concentration of the immersion bath. From Figure 6a, we could observe a significant effect of the immersion bath salinity on the equilibrium volume swelling ratio of the hydrogel. While the HN-20/0 only swelled until *Q*_V_ = 2.4, the equilibrium volume swelling ratio moderately increased, first, for the HN-20/0.1 and HN-20/0.25 (*Q*_V_ = 2.5 and 2.6, respectively), then increased further for the HN-20/0.5 (*Q*_V_ = 2.9) to reach a *Q*_V_ value as high as 4.0 for the HN-20/1 which is close to the one of the pure pAAm hydrogel SN-0 (*Q*_V_ = 4.5). Thereafter, the HN-20/X samples were subjected to uniaxial tensile testing until failure (Figure 6b) and cyclic loading–unloading (Figure 6c). Figure 6b shows that upon increasing the salinity of the immersion bath, the stretchability of the hydrogels increased simultaneously as their ultimate stress and stiffness decreased gradually (Figure S7a–d). In addition, Figures 7c and S8a,b show the gradual decrease of the residual strain as well as the hysteresis area between the loading and unloading curve, highlighting the reduction in toughness and plasticity of the HN-20/X samples as the salinity of the immersion bath was increased up to 1 M. Remarkably, the Young’s modulus, the ultimate stress and strain, and the toughness value of the HN-20/1 all closely matched the values determined for the pure pAAm, SN-0 hydrogel. These results demonstrate that the salt-induced plasticization of PEC hydrogels is still possible when interpenetrated with the pAAm matrix. The evidence strongly suggests that it is possible to tune the stiffness, plasticity, and toughness of the resulting material in the same way as for pure PEC hydrogels.

## 4 Discussion

Figure 7 shows a schematic picture of how the granular hybrid network architecture is obtained during the synthesis (Figure 7a) and the contributions of both networks on the observed mechanical properties (Figure 7b). First, the system starts as a homogeneous mixture of monomers, polyelectrolytes, and salt, at concentrations above the critical concentration for complex coacervation. Upon polymerization and concomitant cross-linking of the pAAm chains, an additional entropic contribution from these neutral water-soluble chains leads to demixed pSS-pDADMAc droplets within the pAAm matrix. This LLPS induced by the “crowding” of the pAAm chains is limited to a few hundred nanometers or several micrometers as the HN hydrogels remain homogeneously turbid for a prolonged time (at least 1 week). The thermodynamics and kinetics of the LLPS inside an elastic gel matrix have already been studied for a few other systems (temperature-induced demixing of a fluorinated oil in a silicone gel,^55^ formation of anise oil-rich droplets inside a hydrogel matrix through diffusion of excess water inward the hydrogel). These studies highlight the critical effect the stiffness of the matrix has on limiting the coalescence and ripening of the dispersed droplets.^56−60^ Upon desalting, the results suggest that the electrostatic complexation between pSS and pDADMAc is enhanced and that these previously formed liquid PEC droplets harden and deform to merge into a rigid granular PEC network interpenetrated within the pAAm network. The higher the PEC content, the stronger it is in preventing the pAAm network from getting hydrated and swelling. This intricate interplay between the two networks leading to this peculiar microarchitecture is also decisive when interpreting the mechanical properties and the dimensional stability of the resulting HNs schematized in Figure 7b. In classical cyclic loading–unloading testing, the pristine HN hydrogel is stretched from its original dimension *l*_0_ to 1.5 *l*_0_ corresponding to a strain of 0.5 mm.mm^–1^. Upon stretching, energy is dissipated in the hydrogel due to the “unzipping” of the electrostatically associated chain segments of pSS and pDADMAc. Immediately upon release of the stress, due to the strong and thermodynamically favored electrostatic associations between the polyelectrolyte chains, the HN hydrogels exhibit an increased residual deformation when increasing the PEC content (*l*_r_). This fast electrostatic reassociation is marked by the instantaneous recovery of almost 50% of the toughening potential of the PEC network in the HN-20 hydrogel. Concerning the remaining 50%, rather than irreversibly broken associations, the originally mechanically active associated polyelectrolyte pairs reassociate at a new equilibrium position where they are not activated during a second loading cycle to an identical strain of 0.5 mm.mm^–1^. Upon rest, we observed that all the HN-X hydrogels recovered their original dimensions (*l*_0_) in less than 300 s, in striking contrast with PEC-20 hydrogel, which exhibited more than 0.25 mm.mm^–1^ residual strain after 500 s. The strong intrinsic rubber elasticity of the covalent pAAm network ensures this fast residual strain recovery. Thus, the cross-linked pAAm chains provide a repelling force on the PEC network, restoring the original dimensions of the samples. This repelling mechanism also contributes to the enhanced toughening self-recovery efficiency of the HN-20 hydrogel with time which reaches a steady value of 80% also after 300 s rest as the associated polyelectrolyte segments are pulled back within a zone in the sample where they can be activated before stretching it to 0.5 mm.mm^–1^.

Finally, one point that is important to bring to the attention of the reader is the reasonably low toughness of our HN hydrogels compared to that of the reported double network hydrogels based on a supramolecular sacrificial network. Indeed, the strain energy density measured for this type of double network hydrogel usually reaches 1 000 and 10 000 kJ mol^–1^. In contrast, our HN-20 hydrogel reaches a value of around 225 kJ mol^–1^ (Figure S9b).^8,9,53,54,61^ We hypothesize that the formation of the phase-separated PEC granules within the hydrogel drastically hampers the necessary stress delocalization along this network upon stretching. The chains of the pAAm are, to some extent, trapped between the granules leading to an accumulation of the stress through the network that dramatically breaks at an early stage (around 1.5 mm.mm^–1^ strain) and provokes the macroscopic failure of the hydrogels. Consequently, we suppose that the “crowding effect” due to the pAAm network polymerization is the original cause of this low toughness observed. From this hypothesis, and building on the knowledge obtained from the molecular dynamics simulation of the LLPS process happening in the HN hydrogels, we can propose two study tracks that appear to be contradictory at first glance, but which target the same goal: keeping the PEC network the most molecularly dissolved possible within the pAAm matrix. (1) The first track uses shorter polyelectrolyte chains to lower the two-phase boundary toward decreasing salt concentrations, as commonly reported for complex coacervates. In that case, even in the presence of “crowding”, the pSS and pDADMAc would remain dissolved until their electrostatically driven assembly is triggered by desalting the hydrogel. (2) The second track becomes relevant if the LLPS cannot be prevented by using shorter polyelectrolyte chains. In that case, a completely reversed approach would rely on using longer chains than the ones used for our study. Indeed, the molecular dynamics simulation reported in Figure S7 points out a favorable effect of increasing the size of the chain on limiting the extent of LLPS within the growing pAAm hydrogel matrix. This is explained by the higher density of entanglements with the pAAm network that can be reached using longer chains, which would kinetically prevent dramatic phase separation.

## 5 Conclusions

In summary, we demonstrated a new way of preparing tough hybrid network hydrogels with a complex microarchitecture by performing the desaltation of an initially homogeneous pSS-pDADMAc-KBr solution in an elastic hydrogel matrix. First, the polymerization of the polyacrylamide matrix led to the formation of phase-separated pSS-pDADMAc liquid droplets due to a so-called “crowding” effect arising from an entropic phenomenon driven by the dehydration of the polyelectrolyte chains. When immersed in a salt-free bath, the pSS-pDADMAc droplets solidified into granules to give rise to an interpenetrated hybrid network architecture with the polyacrylamide network. The combination of both networks was found to be efficient in imparting a dissipative mechanism to enhance the toughness of the polyacrylamide network and self-recovery behavior but also to increase the elasticity of the pSS-pDADMAc network by drastically decreasing the residual strain after cyclic loading–unloading. The strategy introduced here can reveal interesting applications for biological tissue mimics or soft optical material purposes, for which hydrogels with complex architecture, where internal inhomogeneities with different characteristic length scales are advantageously used. Additionally, we envisage that the high strength of the hybrid network hydrogels originating from the pSS-pDADMAc network can be helpful for use as shape-memory materials, favor their resistance to drying (freeze-drying), and open the way to further modification of the hydrogels in a dry state. Our current work focuses on functionalizing the hydrogels with a conductive polymer coating via oxidative chemical vapor deposition to fabricate soft sensors for biomedical applications. From the simulation or experimental perspective, future work could systematically investigate how different tunable parameters, such as polymer network concentration or cross-linking density, would affect the crowding phenomenon and the final double network structure.

## Supporting Information Available

^1^H NMR of the pSS-pDADMAc mixture, dimensions of the dog-bone shaped samples, TEM image and EDX of the HN-5 hydrogel, MD simulation of the crowding effect, ESEM images of the hydrogels surface and cross-section, MD simulation of the desalting process, strain energy density, ultimate strength, and ultimate strain of the SN-0, HN-X, and PEC-20 hydrogels, Young’s modulus, strain energy density, ultimate strength, ultimate strain, energy dissipated and residual strain of the HN-20 hydrogel in bath with different salinity, and composition of the hydrogels precursor solutions (PDF)
