# Supplementary material for: Structure–Property Relationships of Granular Hybrid Hydrogels Formed through Polyelectrolyte Complexation
Source: Macromolecules. 2024 Mar 20;57(7):3190–201. doi: 10.1021/acs.macromol.3c02335 (PMC11008357; doi:10.1021/acs.macromol.3c02335)
Supplement: Supplementary file 1 — ma3c02335_si_001.pdf [file ma3c02335_si_001.pdf]

Structure-Property Relationships of Granular Hybrid Hydrogels formed through Polyelectrolyte Complexation

Julien Es Sayed[a*], Adrivit Mukherjee[a,b], Siham El Aani[a], Nayan Vengallur,[a], Marcus Koch[c], Andrea Giuntoli[a*], Marleen Kamperman[a*]

[a]*Zernike Institute for Advanced Materials (ZIAM), University of Groningen, Nijenborgh 4, 9747 AG Groningen, the Netherlands.*

[b]*Engineering and Technology Institute Groningen (ENTEG), University of Groningen, Nijenborgh 4, 9747 AG Groningen, The Netherlands*

[c]*INM – Leibniz Institute for New Materials, Campus D2.2, Saarbrücken 66123, Germany*

*E-mail : j.s.es.sayed@rug.nl, a.giuntoli@rug.nl, marleen.kamperman@rug.nl

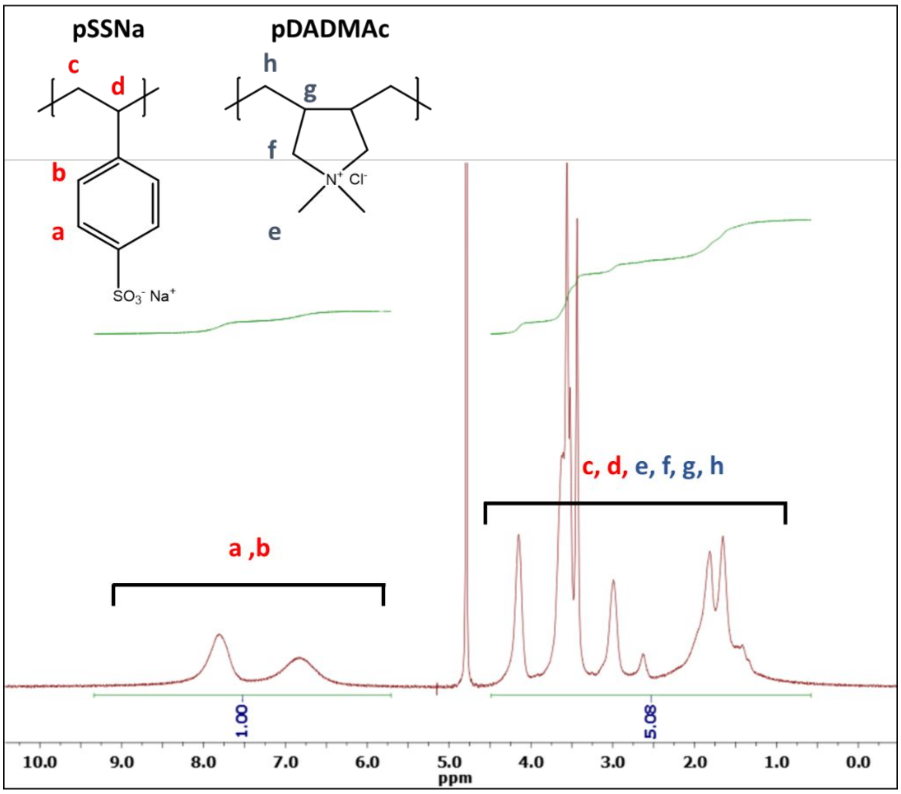

Figure S1. ^{1}H NMR spectrum (D_2O + 2.5M KBr) of the pSS-pDADMAc mixture. The negative to positive charge ratio was calculated as follows: (-)/(+) = $\frac{4A_{a,b}}{4A_{c,d,e,f,g,h} - 3/4A_{a,b}}$, where A_x is the integrated area of the proton x.

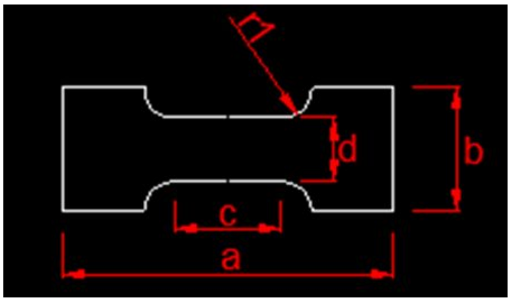

a (mm)	b (mm)	c (mm)	d (mm)	r1 (mm)
40	16	12	5	5

Figure S2. Dimensions of the dog-bone shaped samples.

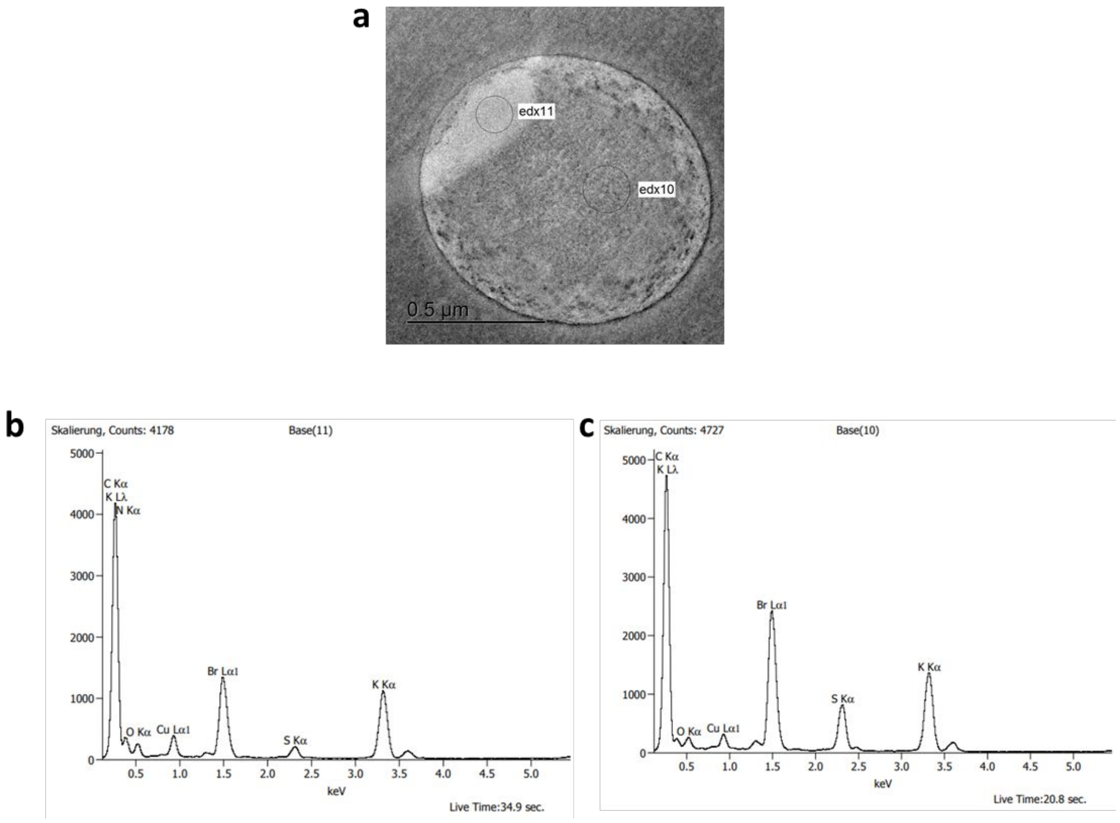

Figure S3. (a) Transmission electron microscopy image of the HN-5 hydrogel. Energy-dispersive X-ray spectroscopy (EDX) analysis of the **(b)** granule-free area (edx11) and **(c)** granules-rich area (edx10).

Determination of the critical salt concentration

We determined the critical salt concentration necessary to prevent the polyelectrolyte complexation by removing the neutral particles from the original system and progressively adding salt ion beads until no coacervation is observed. This corresponds to an unchanged polyelectrolyte surface area when the charged interactions are enabled after equilibration. In the **Figure S4**, we can observe that 1.7 10^5 ion beads are necessary to prevent complex coacervation.

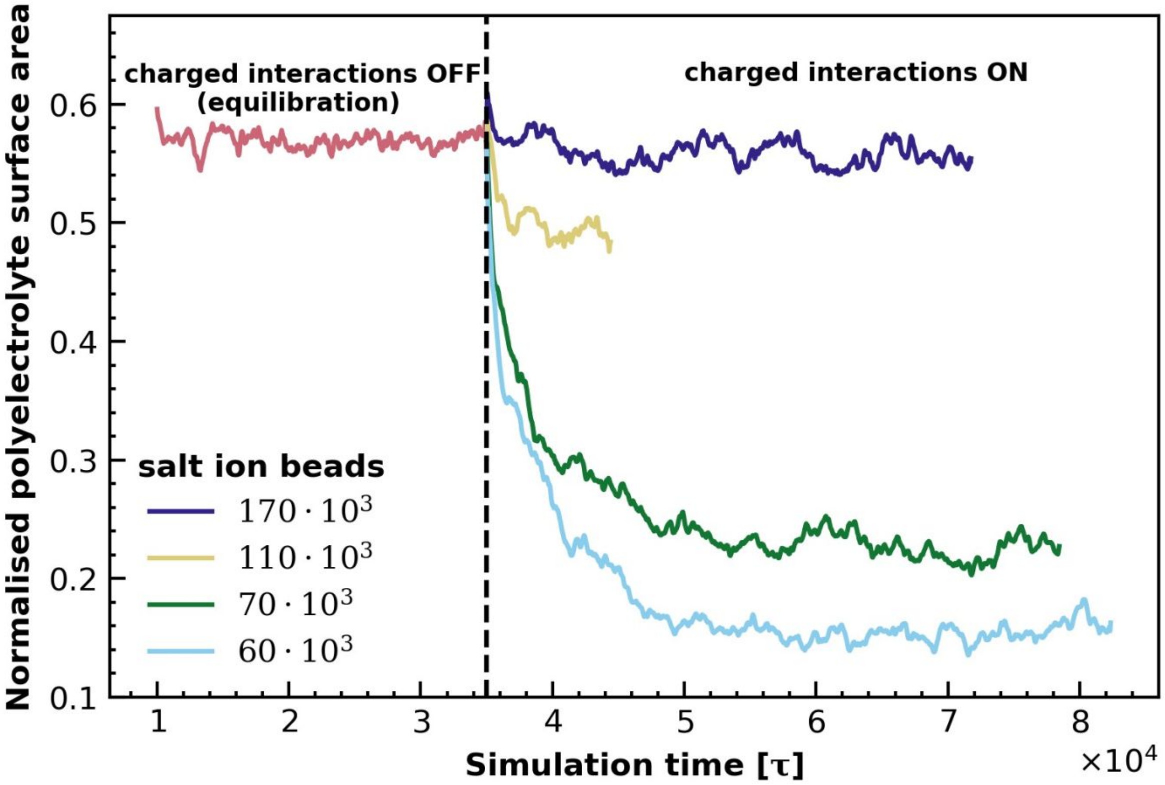

Figure S4. Normalised polyelectrolyte surface area of the system with no pAAm beads and varying amount of salt ion beads up to the critical salt concentration (1.7 10^5 salt beads). The extent of complexation is proportional to the decrease in surface area upon the activation of charged interactions. The critical salt concentration is reached when no reduction is observed.

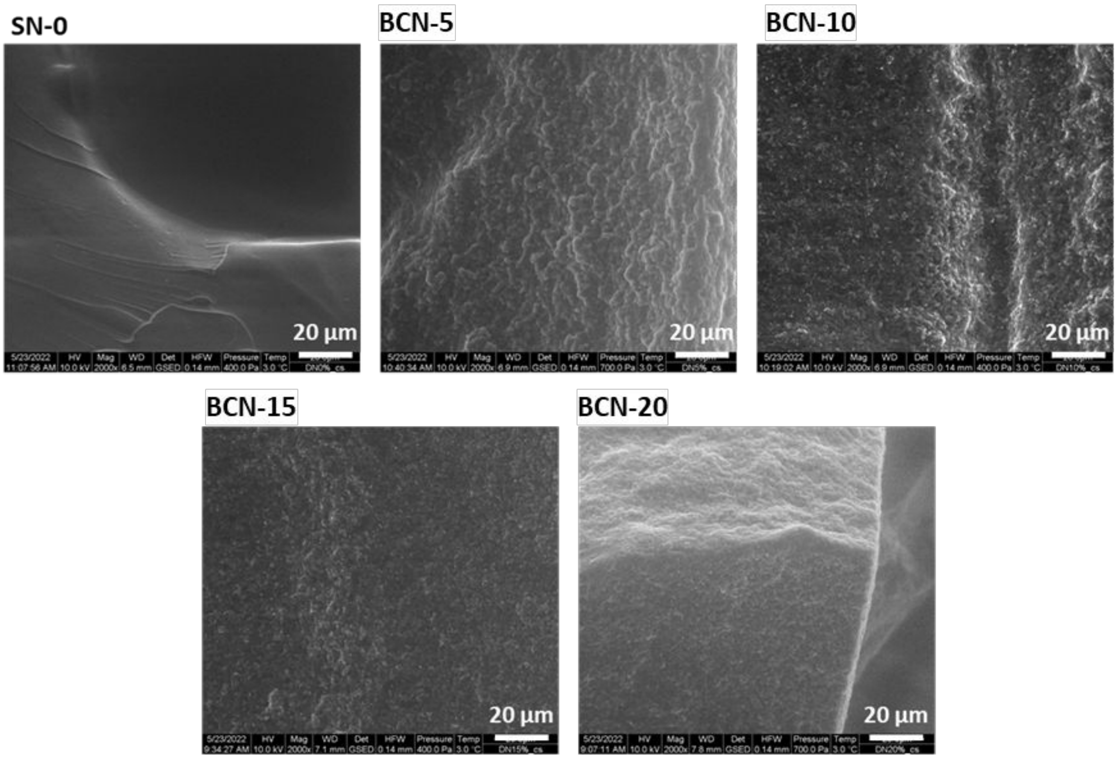

Figure S5. Environmental scanning electron microscopy (ESEM) images of the SN-0, HN-5, HN-10, DN-15 and HN-20. The scale bars are 20 µm.

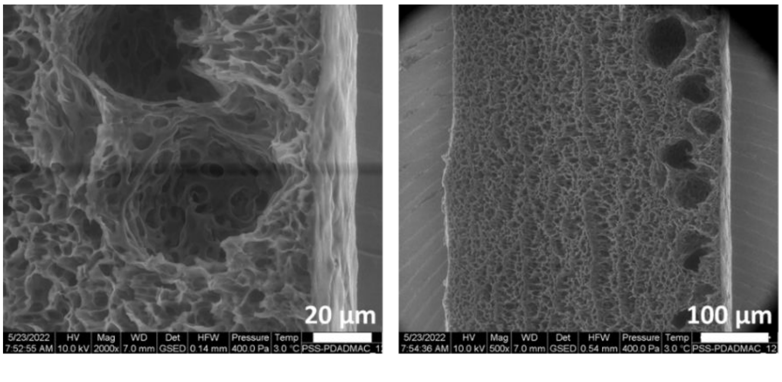

Figure S6. Cross-section images obtained by ESEM of the PEC-20 hydrogel. The dense skin can be observed on the right hand side of the cross-section.

Network formation in the entanglement regime

The absence of a kinetic arrest observed in the short scale simulations suggests that the system is not operating within the entanglement regime. To confirm this hypothesis, we conducted simulations of the desalting process using polymer chains with higher molecular weight and implicit salt. In these simulations, the new polyelectrolyte chain length was set to 200, with a star arm length of 30.

Initially, we carried out equilibration and crosslinking steps without any charged interactions. Subsequently, we turned on the electrostatic interactions while setting the dielectric constant to 10 to account for the screening effects induced by salt ions. To facilitate rapid desalting, we reduced the dielectric constant to 1 over 250 τ and the system was simulated until a steady state is reached. We repeated the same procedure on the original system for comparison.

We observe a sudden drop in the polyelectrolyte area when the charges are turned on. Following this, the system with short chains completely phase separates, while the larger system shows kinetic arrest of the polyelectrolyte chains.

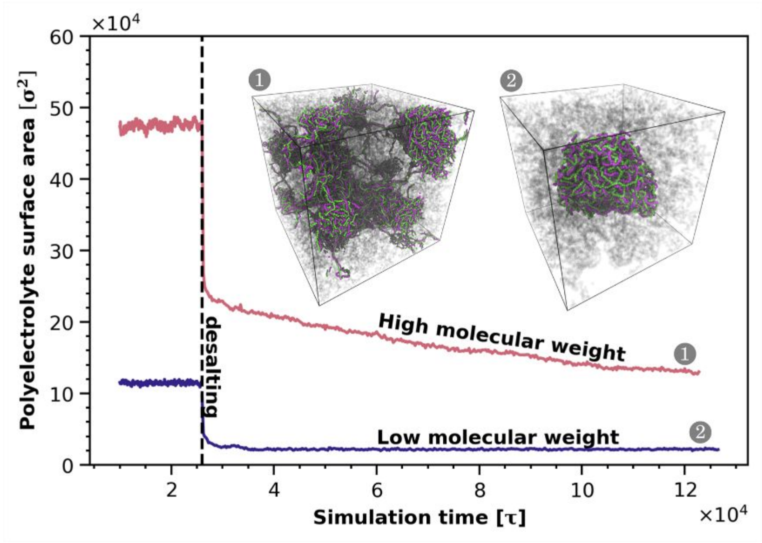

Figure S7. Polyelectrolyte surface area during the desalting phase. The system with high molecular weight shows kinetic arrest, while the smaller system completely phase separates, even tough the ratio of polyelectrolyte chain length and length of the strands between crosslinking sites of the polyacrylamide remains constant.

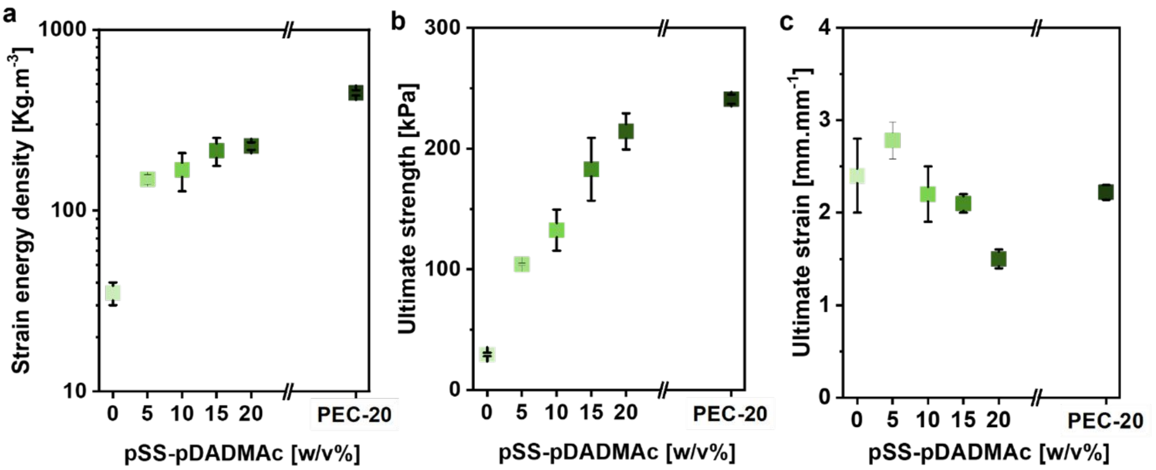

Figure S8. Evolution of the **(a)** strain energy density, **(b)** ultimate strength and **(c)** ultimate strain of the SN-0, HN-X and PEC-20 hydrogels as a function of the pSS-pDADMAc content.

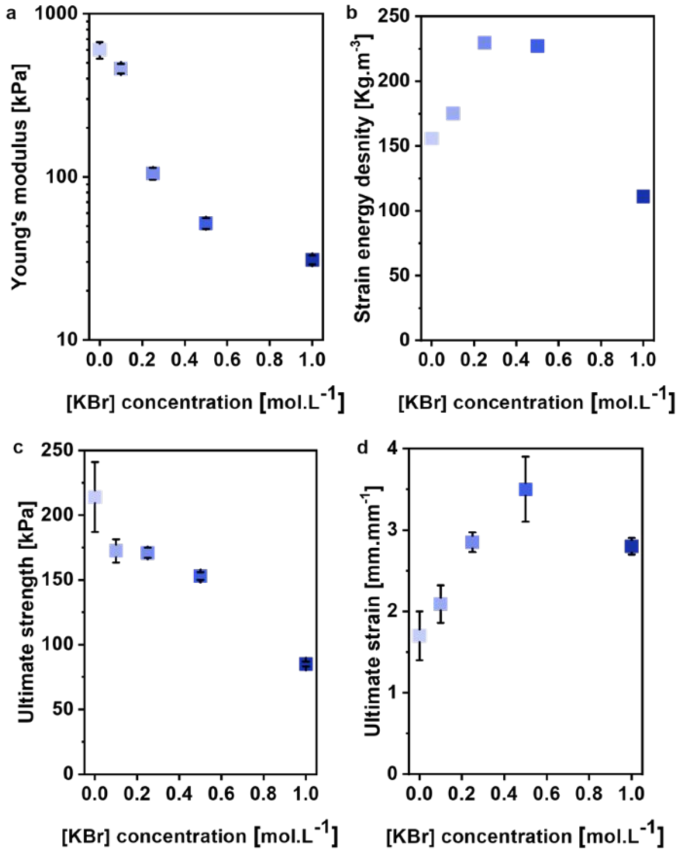

Figure S9. Evolution of the **(a)** Young's modulus, **(b)** strain energy density, **(c)** ultimate strength and **(d)** ultimate strain by tensile testing until failure of the HN-20 hydrogel as a function of the immersion bath salinity.

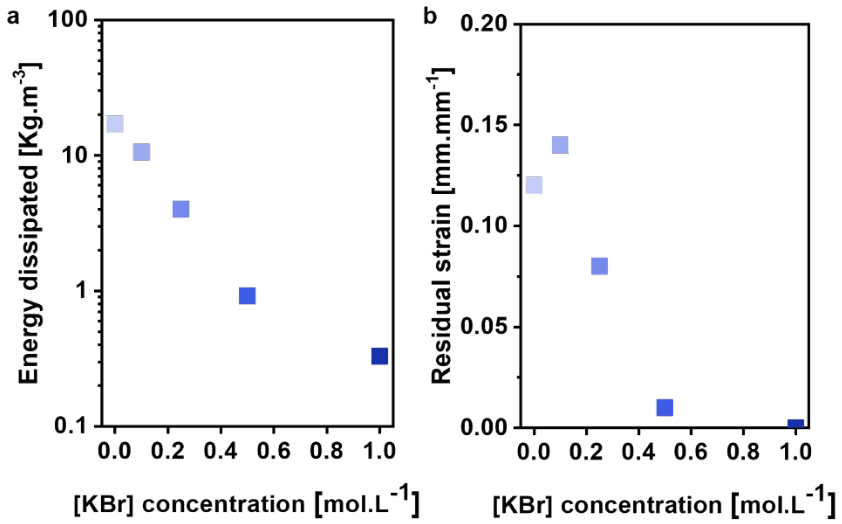

Figure S10. Evolution of the **(a)** the energy dissipated and **(b)** residual strain by cylic tensile testing of the HN-20 hydrogel as a function of the immersion bath salinity.

Table S1. Composition of the precursor solutions of the SN-0 and HN-X hydrogels.

	Volume pSS-pDADMAc 20wt/v% (mL)	Volume 2.5 M KBr water (mL)	AAm (g)	MBA (g)	APS (g)	TEMED (mL)
**SN-0**	0	4	1.136	0.0024	0.0022	0.03
**HN-5**	1	3	1.136	0.0024	0.0022	0.03
**HN-10**	2	2	1.136	0.0024	0.0022	0.03
**HN-15**	3	1	1.136	0.0024	0.0022	0.03
**HN-20**	4	0	1.136	0.0024	0.0022	0.03
